# Investigation of the Synergistic Effects of Different Salts in Smart Water Injection Fluids on Oil–Brine Interfacial Tension

**DOI:** 10.1021/acsomega.5c00851

**Published:** 2025-09-30

**Authors:** Pamela D. Rodrigues, Cristina M. Quintella, João Pedro D. Rodrigues, Larissa S. de S. Figueiredo, Jorge L. Nicoleti, Edgard B. Carvalho, Elias R. de Souza, Samira A. Hanna

**Affiliations:** † Institute of Chemistry, Federal University of Bahia, Ondina Campus, Rua Barão de Jeremoabo, s/n, 40170-115 Salvador, Bahia, Brazil; ‡ Interdisciplinary Center for Energy and Environment, Federal University of Bahia, Ondina Campus, Rua Barão de Jeremoabo, s/n, 40170-115 Salvador, Bahia, Brazil; § Institute of Health Sciences, Federal University of Bahia, Avenida Reitor Miguel Calmon, s/n, Vale do Canela, 40110-100 Salvador, Bahia, Brazil; ∥ 169704 Federal Institute of Education, Science and Technology of Bahia, Salvador Campus, Rua Emídio dos Santos, s/n, Barbalho, 40301-015 Salvador, Bahia, Brazil; ⊥ Fluid Mosaic Research and Innovation Ltd, Avenida Manoel Dias, s/n, 41830-000 Salvador, Bahia, Brazil

## Abstract

Although the need for an energy transition is increasingly evident, fossil fuels will remain essential for humanity, highlighting the necessity for responsible migration strategies. In this context, low-salinity injection methods enable smart management of oil production while addressing the United Nations Sustainable Development Goals (SDG 7) in the 2030 Agenda. These methods reduce the environmental impact of oil production while ensuring the continuation of fossil fuel extraction necessary for social well-being in the coming decades. Despite the absence of consensus on the optimal saline composition and concentrations in smart water, its effectiveness is largely linked to changes in wettability and reductions in interfacial tension (IFT) within the oil–brine–rock system. This study explores the synergistic effects of sodium chloride, calcium chloride, and sodium bicarbonate across two total dissolved solids ranges (0–70,295 mg/L and 2200–24,813 mg/L) on IFT with Brazilian presalt oil, comparing the results with oil recovery factors obtained from carbonate rock tests. The findings indicate that salinity significantly influences the individual effects of each salt on IFT. In high-salinity scenarios, the presence of CaCl_2_ notably increases IFT with oil, suggesting that the bilayer effect between Ca^2+^ ions and organic acids predominates. Conversely, at low salinity, NaCl demonstrated the most significant impact in reducing the IFT, indicating that the salting-in phenomenon prevails. The most pronounced synergistic effects occurred between CaCl_2_ and NaHCO_3_ in high-salinity environments and between NaCl and NaHCO_3_ in low-salinity situations. In both cases, the interference was positive, suggesting that the deprotonation of organic acids facilitated by the basic salt does not aid in lowering the IFT when combined with other salts. However, in high-salinity environments, NaHCO_3_ exhibited negative interference but contributed to a reduction in IFT. A correlation was observed between the decrease in the IFT and an increase in the oil recovery factor, particularly under conditions with high NaCl concentrations and low to medium levels of CaCl_2_ and NaHCO_3_.

## Graphical abstract

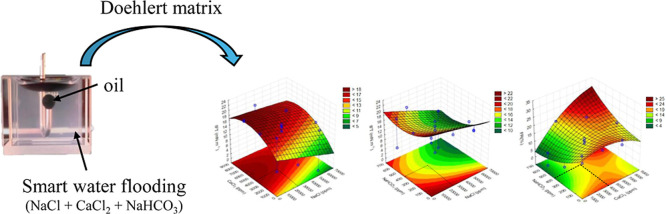

## 1 Introduction

The need for more sustainable energy alternatives is increasingly urgent; however, fossil fuels will remain essential in the coming decades. Therefore, minimizing environmental impacts during their production is crucial.^1^ One of the most promising techniques for enhanced oil recovery (EOR) in carbonate reservoirs is smart water flooding (SWF).^2,3^ Smart water brines possess a specific ionic composition that enhances oil recovery.^4^ They do not require costly additives, are free from injection issues, and are generally environmentally friendly.^5,6^

The mechanisms of EOR through SWF, and their dependence on the concentration and types of salts used, remain unclear. Generally, most researchers agree that SWF injection impacts the wettability and interfacial tension (IFT) of the oil–brine–rock system.^7–10^ The IFT between oil and the injection fluid is a critical factor in the oil recovery process,^11^ as it is directly linked to the capillary forces acting on trapped oil and its potential for mobilization.^12^ IFT can be reduced by various factors, including pH conditions, mineral dissolution, emulsification, and saponification—all of which are directly related to wettability alteration.^6^

Understanding the effect of brine ionic composition on IFT is crucial for the application of SWF in EOR. Numerous experimental and theoretical investigations have aimed to elucidate the impact of salinity on oil–brine IFT.^13–16^ However, the findings often present more contradictions than consistent conclusions about the subject.

Most existing studies do not consider complex compositions in the brine or oil, focusing instead on the effects of monovalent and divalent salts on IFT separately. This approach overlooks the potential synergistic effects of the interacting ions that can influence the oil–brine interface. Consequently, the results of these studies are often diverse and even contradictory.

Kakati et al. (2017)^17^ conducted IFT measurements between brines containing NaCl, CaCl_2_, and MgCl_2_ at concentrations ranging from 0 to 30,000 ppm in both aromatic and aliphatic hydrocarbons. They observed that the type of hydrocarbon influences the IFT and identified a critical salinity for each salt that results in a maximum IFT reduction. Notably, NaCl, as well as CaCl_2_ and MgCl_2_, was found to be particularly effective in reducing IFT—especially for aliphatic hydrocarbons.

Bai et al. (2010)^18^ investigated the effect of NaCl at concentrations ranging from 0 to 10,000 ppm on IFT with crude oil and its polar fractions (asphaltenes and resins). They observed that within the studied concentration range, NaCl had no significant impact on IFT.

Honarvar et al. (2020)^7^ separately examined the effects of monovalent salts (NaCl and KCl) and divalent salts (CaCl_2_, MgCl_2_, and Na_2_SO_4_) on IFT with crude oil at concentrations ranging from 0 to 120,000 ppm. They concluded that each salt has an optimal concentration that minimizes IFT, with CaCl_2_ and NaCl being the most effective salts in reducing the IFT.

Lashkarbolooki et al. (2017)^5^ conducted IFT measurements between brines containing NaCl, CaCl_2_, and MgCl_2_ at concentrations ranging from 0 to 45,000 ppm with crude oil and its polar fractions. They demonstrated that while NaCl reduces IFT, divalent salts are more effective in this reduction. However, few studies have explored the synergistic effects of the salts present in brine.

Abdel-Azeim et al. (2021)^11^ identified a synergistic effect among various ions in high-salinity brine, as well as a specific interaction between organic acids and Ca^2+^ at the oil–brine interface. In a complex solution, Ca^2+^ ions become encapsulated within the brine, rendering them unavailable for interaction with the oil, which leads to an increase in IFT, an effect not observed in individual brine solutions.

For EOR, Sohal et al. (2017)^19^ conducted a multivariate analysis using principal component analysis (PCA) on bibliographic data. While they could not determine the best mechanisms, they concluded that for carbonate reservoirs, low-salinity water (smart water) containing both divalent cations (Ca^2+^, Mg^2+^) and monovalent cations (Na^+^) is beneficial. Quintella et al. (2023)^1^ further identified that maximizing the recovery factor requires synergies between monovalent and divalent salts.

Based on these inconsistencies, this study hypothesizes that IFT is not determined by the concentration of individual salts alone but by the synergistic interactions between specific ions in the brine and the complex molecular structure of the crude oil. Furthermore, we hypothesize that certain ion combinations—particularly involving Na^+^, Ca^2+^, and HCO_3_^–^—result in enhanced IFT reduction due to favorable ion–ion and ion–oil interactions.

Given the diversity and inconsistency of results and proposed mechanisms in the literature on SWF applications,^19^ evaluating IFT is essential for clarifying the mechanisms of fluid–fluid interaction in oil recovery. This study combines the Doehlert experimental design with the hanging drop method to analyze the oil–brine IFT, considering the complex composition of Brazilian presalt crude oil and three salts: sodium chloride (NaCl), calcium chloride (CaCl_2_), and sodium bicarbonate (NaHCO_3_) from SWF formulations. The aim is to investigate the possible synergistic effects of interparticle interactions—ion/ion and ion/oil—seeking a deeper understanding of the correlation between salinity and IFT. This approach aims to optimize formulations to reduce IFT, thereby improving oil mobility in EOR applications.

## 2 Methodology

### 2.1 Characterization of Petroleum

The crude oil used in the experiments is sourced from the Jupiter/RJ field in the Brazilian presalt region and has an API gravity of 28.

SARA analysis (saturates, aromatics, resins, and asphaltenes) is a method that categorizes the components of crude oil into groups based on their polarizability and polarity.^20^

Based on ASTM D3279 and D2007 standards, this analysis was performed through solvent fractionation of a petroleum sample using column chromatography, followed by gravimetric determination of the content of each group after solvent evaporation. The results are presented in Table 1.

**Table 1:** SARA Compositional Analysis and TAN of the Crude Oil

SARA fractional analysis	
group	wt (%)
saturates	63,2
aromatics	18,8
resin	17,8
asphaltene	0,18
total acid number (TAN) = 1.01 mg KOH g^–1^ oil	

Another parameter measured for the crude oil sample was the total acid number (TAN). This parameter serves as a good indicator of the surface activity of the chemical components present in oil.^13^ Crude oil is considered acidic if the measured TAN exceeds 0.5 mg KOH g^–1^ of crude oil.^21^ Therefore, the TAN of the crude oil was measured using the ASTM D 974 standard test method through volumetric acid–base neutralization titration.^22,23^

### 2.2 Design of Experiments

To develop a mathematical model and response surfaces that describe the behavior of oil/smart water IFT in relation to the saline composition of sodium chloride (NaCl), calcium chloride (CaCl_2_), and sodium bicarbonate (NaHCO_3_), as well as any potential synergistic effects, the Doehlert experimental design methodology was employed, incorporating a repetition at the central point. This approach follows a previous study that established a correlation between the composition of similar smart waters and the recovery factor (% RF).^1^

In Doehlert experimental design, it is possible to analyze two, three, or more independent variables using a reduced number of tests compared to other experimental methodologies, without compromising the quality of the obtained responses.^24^ In this study, the independent variables were the concentrations (in parts per million) of the salts NaCl, CaCl_2_, and NaHCO_3_ present in the SWF solutions, while the response variable was the IFT with oil.

As a first strategy, a broader range of salinities was explored. Subsequently, a second strategy was designed to identify the effects of these salts in a low-salinity region. Table 2 presents the minimum, average, and maximum parameters used to generate the Doehlert matrix for each strategy. For each strategy, 15 formulations were developed, including a repetition at the central point, resulting in a total of 30 different saline solutions.

**Table 2:** Values Defined for the Independent Variables of the Doehlert Matrix

	first strategy—wide salt			second strategy—low salt		
salt	minimum value	average value	maximum value	minimum value	average value	maximum value
NaCl (ppm)	0.0	25,000	50,000	5000	10,000	20,000
CaCl_2_ (ppm)	0.0	3000	6000	1000	2000	3000
NaHCO_3_ (ppm)	0.0	250	500	100	200	300

The experimental values obtained for the IFT of each SWF formulation were statistically analyzed using Statistic software with multiple regression. This analysis allowed for the identification of factors that influence the responses linearly as well as their interactions. Multiple regression was conducted using coded independent variables to standardize the actual factor values, ensuring that the statistical analyses were not adversely affected by the diverse range of factor values. An analysis of variance (ANOVA) was performed to assess the statistical significance of the mathematical model derived from multiple regression. A confidence level of 95% was adopted for the statistical analyses.

### 2.3 IFT Measurements

All IFT measurements were conducted using dead crude oil at atmospheric pressure. While it is acknowledged that IFT behavior can differ under reservoir conditions, particularly due to the presence of live oil and elevated pressures, the use of dead oil under ambient conditions remains a widely accepted approach for comparative and preliminary screening purposes. These conditions allow for consistent, reproducible measurements that provide valuable insights into fluid–fluid interactions.^25^ Moreover, the fundamental interfacial trends observed are still indicative of the system’s behavior and can inform further high-pressure studies.

IFT measurements between dead crude oil and SWF solutions were conducted using the pendant drop method on a DataPhysics tensiometer, model OCA 15 plus. The equipment is equipped with a temperature controller, and measurements were taken at 65 ± 2 °C. Each SWF solution was placed in a quartz cuvette, and an oil droplet was formed within the solution using an inverted needle. A high-resolution camera captured images of the droplets, and built-in software analyzed the droplet dimensions to calculate the IFT using the Young–Laplace equation. Five well-formed oil droplets were analyzed for each solution, and the average IFT was used as the response value.

### 2.4 Recovery Factor Measurements

Recovery factor measurements were carried out on carbonado plugs in the Holder system using the same oil and the same SWF solutions tested in the IFT measurements. The methodology and full results were previously published by Quintella et al. (2023).^1^

## 3 Results and Discussion

The compositions of the three salts and the IFT values found for each SWF solution are listed in Table 3. The recovery factors (RF %) listed were obtained from previously published work.^1^

**Table 3:** Smart Water Composition, IFT, and % RF of the First and Second Strategies

fist strategy—wide salt							second strategy—low salt							
test	NaCl (ppm)	CaCl_2_ (ppm)	NaHCO_3_ (ppm)	pH	IFT (mN m^–1^)	RF %^a^	test	NaCl (ppm)	CaCl_2_ (ppm)	NaHCO_3_ (ppm)	pH	IFT (mN m^–1^)	RF %^a^	
01	0	0	0	6.8	21.4	41.4	17	0	2000	200	7.2	20.3	49.5	
02	0	0	500	9.8	8.3	46.5	18	5000	1000	100	7.7	21.1	44.6	
03	0	3000	250	7.3	18.9	50.7	19	5000	1000	300	7.6	21.75	52.5	
04	0	6000	0	7.0	19.6	46.2	20	5000	3000	100	7.4	21.3	36.1	
05	0	6000	500	7.0	20.0	48.2	21	5000	3000	300	6.9	20.3	45.1	
06	25,000	0	250	9.3	7.0	42.6	22	10,000	318	200	8.9	20.5	40.3	
07	25,000	3000	0	7.0	19.0	49.4	23	10,000	2000	32	7.6	21.6	37.6	
08	25,000	3000	250	7.3	17.7	47.3	24	10,000	2000	200	7.5	19.0	43.6	
09	25,000	3000	250	7.2	16.2	45.2	25	10,000	2000	200	7.4	18.6	43.1	
10	25,000	3000	670	7.1	18.2	45.9	26	10,000	2000	368	7.6	19.9	46.6	
11	25,000	8045	250	7.0	19.8	44.9	27	10,000	3682	200	7.4	20.1	35.9	
12	50,000	0	0	7.1	17.0	44.5	28	20,000	1000	100	7.7	14.9	41.1	
13	50,000	0	500	8.5	0.4	44.4	29	20,000	1000	300	7.9	18.7	46.5	
14	50,000	6000	0	7.0	16.7	42.9	30	20,000	3000	100	7.4	18.1	42.1	
15	50,000	6000	500	6.8	16.7	46.7	31	20,000	3000	300	7.5	19.4	45.3	
16	67,045	3000	250	7.1	13.6	47.7	32	22,613	2000	200	7.5	16.9	47.9	

a Adapted from Quintella et al. (2023).^1^

Statistical analysis allowed for the evaluation of the effects of varying salt concentrations on the IFT with oil. The mathematical model demonstrated a good fit and was statistically significant. The relationship between the mathematical model, evaluated through ANOVA, showed a strong correlation between the experimental and predicted values, as illustrated in Figure 1. The regression models showed high accuracy with *R* ^2^ values of 0.906 (first strategy) and 0.928 (second strategy).

**Figure 1:**
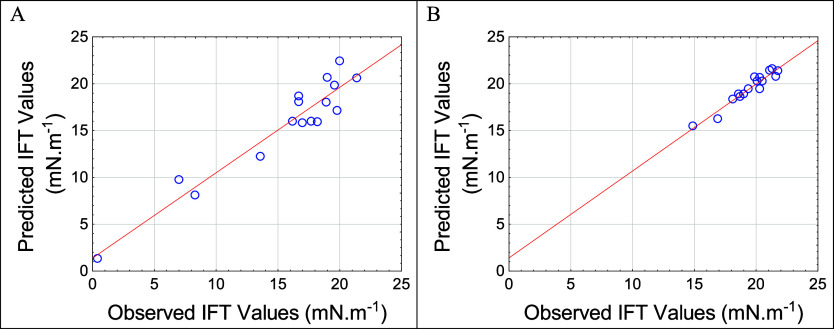
Relationship between experimental results and the mathematical model. (A) Fist strategy; (B) second strategy.

The influence of salts on the IFT with oil for each strategy is presented in hierarchical order in the Pareto diagram, as shown in Figure 2.

**Figure 2:**
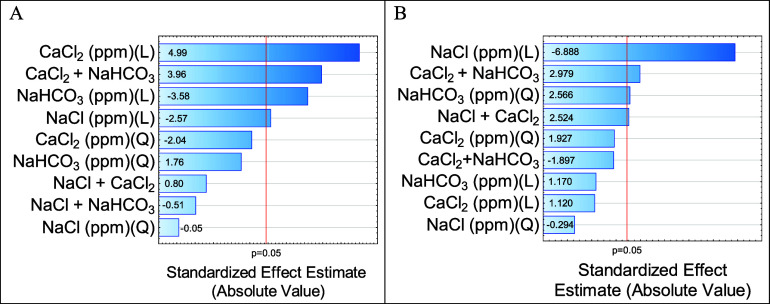
Pareto diagram for the influence of salts on IFT with oil. (A) First strategy; (B) second strategy.

Using the Pareto diagram, it is possible to identify which salts and their combinations have a statistically significant effect (above 5% significance) and whether they exert positive or negative interference on IFT.

The results indicate that the most relevant salts for IFT differ between the two strategies. This suggests that the nature of salt alone cannot fully explain its effects on the IFT; it is also necessary to consider the concentrations of these salts in the solution.

CaCl_2_ was the salt that presented the greatest relevance in the first strategy, but it was not relevant in the second strategy; in addition, it presents positive interference on the IFT in the higher-salinity environment. In other words, in a situation of high salinity, the presence of CaCl_2_ generated a significant increase in IFT. This result corroborates the results found by Abdel-Azeim et al. (2021)^11^ who observed in their simulations that the Ca^2+^ ion presents a strong electrostatic interaction with the organic acids present in petroleum, leading to the formation of a bilayer structure that encapsulates the metallic cations (Ca^2+^), and significantly attenuates the interaction of the organic acid with the phase aqueous resulting in the salting-out effect, leading to a decrease in organic acids at the oil/water interface, generating an increase in IFT. This same effect was not observed in low-salinity or in single-component solutions, which also explains the lack of relevance of CaCl_2_ in a low-salinity environment. Still regarding CaCl_2_, it was possible to observe that even in relevant binary combinations with the others present in Smarts Waters, its interference in the IFT is always positive.

On the other hand, NaCl was the most relevant salt in the second strategy, and it also showed significance above 5% in the first strategy. In both cases, NaCl exhibited negative interference, indicating that its presence in the solution decreases the IFT. This behavior is associated with the salting-in effect as the ions tend to migrate toward the oil–water interface due to their interaction with the polar compounds in petroleum (such as resins and asphaltenes). Consequently, organic components with surfactant properties accumulate at the oil–water interface, thereby reducing IFT.^7^ Although this effect persists in a higher-salinity environment, it is diminished by the stronger attraction between the Ca^2+^ ions and the organic acids present in the oil.

NaHCO_3_ is a basic salt that raises the pH of a solution; however, its application in SWF (surfactant water flooding) solutions has been minimally explored. Nowrouzi et al. (2020)^26^ investigated its effect on reducing IFT in an EOR solution that also contained saponin as a surfactant. Their study demonstrated a reduction in IFT in the range of 0–20,000 ppm compared to a solution with the same surfactant concentration but without saponin.

The results indicate that NaHCO_3_ significantly influenced the IFT, showing a notable effect at concentrations above 5% in the initial strategy. In high-salinity conditions, its presence in the SWF solution decreased IFT. The primary role of this alkaline agent is to react with petroleum acids, which contain active macromolecules with polar functional groups, resulting in the formation of surfactant substances.^27^ These surfactants facilitate the reduction of the IFT between oil and water.

Figure 3 illustrates the IFT response surfaces for the binary combinations of the salts tested, with the third salt held constant at its average concentration. This is compared to the RF response surface for the same binary combinations, adapted from Quintella et al. (2023).^1^ This comparison allows for the visualization of regions where there is a correlation between a higher RF and a lower IFT, or vice versa, in the first strategy.

**Figure 3:**
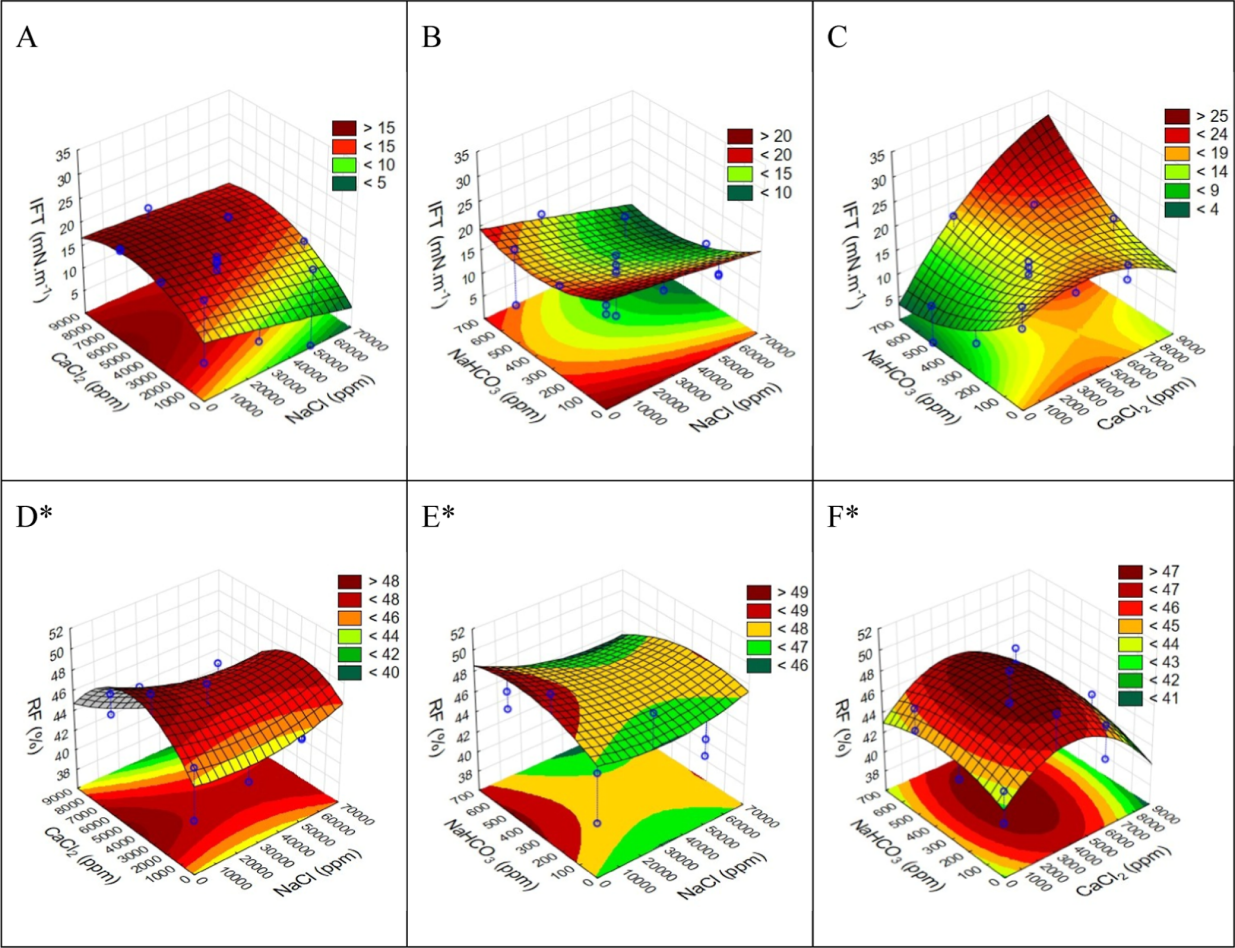
First strategy. (A–C) IFT response surfaces for two-by-two synergic combinations of salts; (D–F) RF response surfaces for two-by-two synergic combinations of salts. *Adapted from Quintella et al. (2023).^1^

Figure 4 presents the IFT response surfaces for the binary combinations of the tested salts, with the third salt held constant at its average concentration. This is compared to the RF response surface for the same binary combinations, adapted from Quintella et al. (2023).^1^ This comparison enables visualization of the regions where a correlation exists between a higher RF and a lower IFT, or vice versa, in the second strategy.

**Figure 4:**
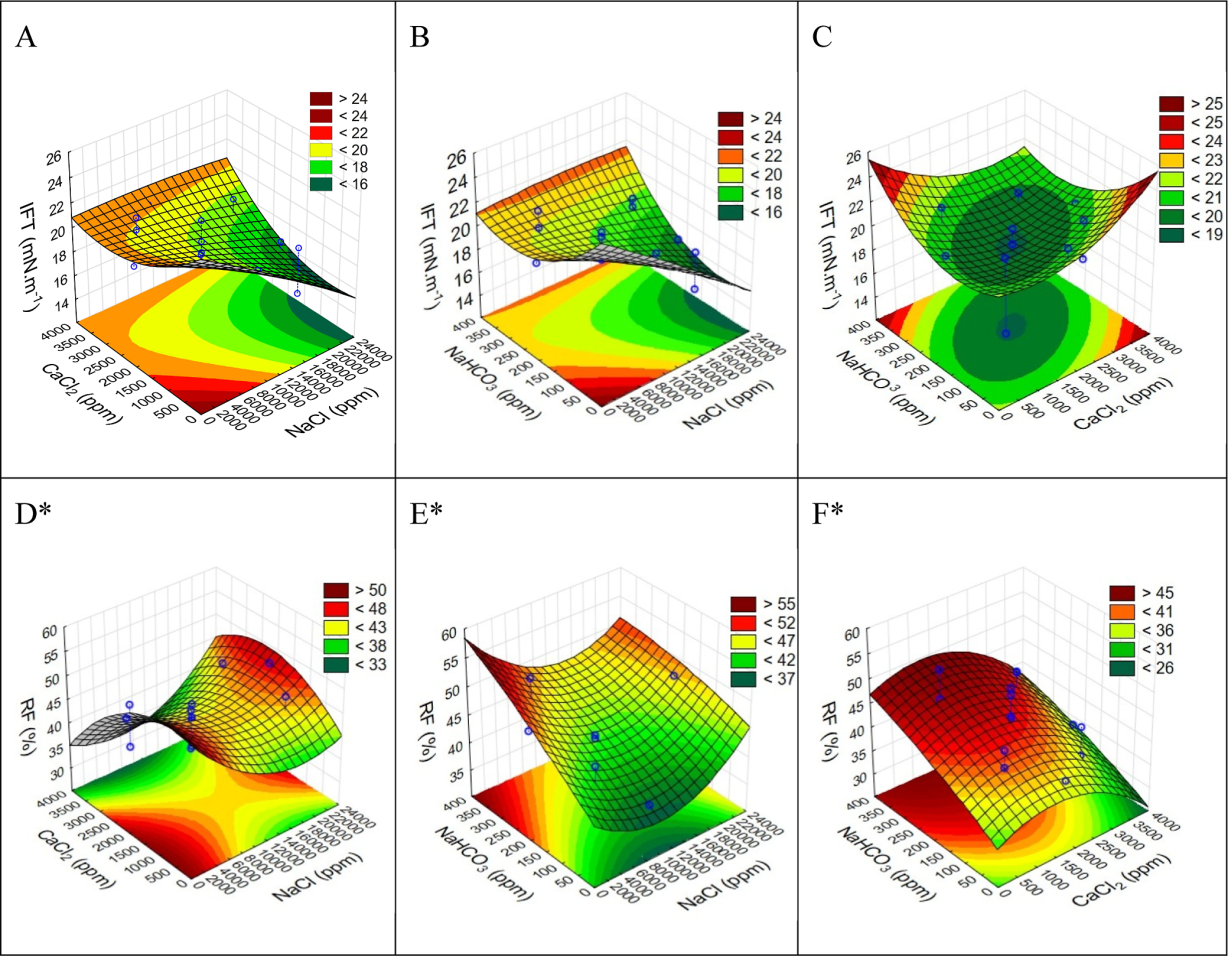
Second strategy. (A–C) IFT response surfaces for two-by-two synergic combinations of salts; (D–F) RF response surfaces for two-by-two synergic combinations of salts. *Adapted from Quintella et al. (2023).^1^

From the response surfaces A–C in Figures 3 and 4, it is evident that while the salinity region of the response surfaces in the second strategy is included within those of the first strategy (marked region), this is not merely an enlargement. This observation indicates that both the nature of the salts and their concentration, as well as their synergy in SWF, significantly influence IFT.

In a salinity situation, the minimum region for the IFT is different, indicating that the salting effect in this case is more relevant than the nature of the salts presented in the brine. The results obtained may even explain the divergence in the literature, as depending on the salinity region, a certain salt may or may not be relevant in lowering the IFT.

Furthermore, when comparing the IFT and RF response surfaces obtained for the same strategy, it is noticeable that in certain regions, a decrease in IFT corresponds to an increase in the recovery factor, while in other regions, an increase in IFT is associated with a decrease in the recovery factor. These observations are highlighted in Table 4.

**Table 4:** Correlation between IFR and RF Response Surfaces

total salinity	RF	IFT	NaCl	CaCl_2_	NaHCO_3_
high (Figure 3A–D)	high	low	high	low	na
low (Figure 4A–D)	high	low	high	medium	na
high (Figure 3B–D)	high	low	high	na	medium
low (Figure 4B–D)	low	high	low	na	low
high (Figure 3C–E)	low	high	na	high	high
low (Figure 4C–E)	high	low	na	medium	medium

In regions where a direct correlation between IFT and RF is observed, we can conclude that the most significant mechanism for oil recovery, under the studied conditions, is the decrease or increase in IFT, which directly impacts the mobility of oil within the reservoir.^28^ In contrast, in regions where this correlation is not present, other mechanisms may be more relevant, potentially involving fluid–rock interactions such as changes in the wettability of the rock.

The observed IFT behavior can be mechanistically explained by the crude oil’s significant content of acidic and polar compounds (TAN = 1.01 mg KOH/g; resins = 17.8 wt %), which are prone to interact with injected brine ions. The strong IFT increase in the presence of Ca^2+^ is consistent with electrostatic bridging and bilayer formation involving carboxylic groups, as supported by molecular dynamics simulations and experimental work.^11^ Conversely, Na^+^ promotes the interfacial accumulation of surface-active species by enabling the salting-in effect, reducing electrostatic repulsion and allowing polar components to adsorb at the oil–brine interface.^5,7^ Furthermore, NaHCO_3_ elevates the pH of the brine, facilitating the deprotonation of organic acids and in situ formation of soap-like amphiphiles, which enhances IFT reduction.^13,26^ These findings suggest that it is important to consider that the chemical composition of crude oil plays a critical role in modulating the IFT through complex ion–organic interactions.

## 4 Conclusion

The study of the influence of Smart Water’s saline composition on IFT with Brazilian presalt oil was conducted using a combination of a semiempirical mathematical model of response surfaces and the hanging drop method. This approach enabled the measurement of IFT for 30 modeled Smart Water formulations utilizing an experimental design based on the Doehlert matrix.

The salts selected for the study (NaCl, CaCl_2_, and NaHCO_3_) are cost-effective and have a low environmental impact, aligning with the demand for more sustainable methods in oil production.

It was evident that salinity directly affects the individual impact of each salt on the IFT reduction or increase. The results demonstrated that under high-salinity conditions, the presence of CaCl_2_ significantly increases IFT with the oil, indicating that the bilayer effect between Ca^2+^ ions and the organic acids in the oil predominates in this scenario.

At low salinity, NaCl was the most significant salt for reducing IFT with oil, suggesting that in this context, the salting-in phenomenon prevails over other mechanisms.

The most significant synergistic effects were observed between CaCl_2_ and NaHCO_3_ in the first strategy (high salinity) and between NaCl and NaHCO_3_ in the second strategy (low salinity). In both cases, the interference was positive, indicating that the deprotonation of organic acids promoted by the presence of the basic salt does not aid in lowering the IFT when combined with other salts. However, in a high-salinity environment, NaHCO_3_ exhibited negative interference and contributed to a reduction in IFT.

The decrease in the IFT exhibited a direct correlation with the increase in the oil recovery factor in carbonate reservoirs, particularly in scenarios with high NaCl concentration and low to medium concentrations of CaCl_2_ and NaHCO_3_.

## 5 Future Recommendations

The results obtained in this study indicate that the ionic composition of the injection water significantly influences the oil–brine IFT, particularly due to total salinity and the synergistic interactions between the present ions. Despite the progress made, several knowledge gaps remain and should be explored in future studies:

- Influence of crude oil composition: This study used presalt Brazilian crude oil, whose acidity and molecular structure affect its interactions with ions in solution. Investigations comparing different types of crude oil could broaden the understanding of the applicability of the proposed formulations.
- Wettability mechanisms and rock mineralogy: While IFT reduction is correlated with an increase in the oil recovery factor in several scenarios, this correlation is not always direct. This suggests that other mechanisms, such as wettability alteration and oil–rock interactions, may be more relevant. Future studies combining IFT and contact angle measurements across various carbonate and siliciclastic lithologies are recommended.
- Effects of temperature and pressure: The experiments were conducted at a constant temperature under atmospheric conditions. Experiments under reservoir-like conditions (high temperature and pressure) are needed to validate the results and assess potential changes in dominant mechanisms.
- Molecular modeling and dynamic simulations: Computational modeling can help elucidate mechanisms such as ion encapsulation, bilayer formation, and their influence on the distribution of interfacial species. Investment in such approaches could complement experimental data and guide the development of more efficient formulations.

These directions point to a research agenda that can not only deepen our understanding of the mechanisms underlying SWF techniques but also support the practical optimization of brine formulations for diverse geological and petroleum production contexts.
